# A qualitative exploratory study of selected physicians’ perceptions of the management of non-communicable diseases at a referral hospital in Zimbabwe

**DOI:** 10.1186/s12992-021-00730-3

**Published:** 2021-07-19

**Authors:** Alexander Cheza, Boikhutso Tlou

**Affiliations:** School of Nursing and Public Health, University of KwaZulu-Natal, 1st Floor, George Campbell Building, Howard College Campus, UKZN, Durban, 4000 South Africa

**Keywords:** Non-communicable diseases (NCDs), Healthcare, Cancers, Diabetes, Cardiovascular disease (CVD), Hypertension

## Abstract

**Introduction:**

Non-communicable diseases (NCDs) have recently become a global public health burden and a leading cause of premature death, mainly in low- and middle-income countries (LMICs). The aim of the study was to explore physicians’ perceptions on the availability and quality of clinical care for the management of NCDs.

**Methods:**

This was a qualitative exploratory study meant to obtain expert perceptions on clinical care delivery for NCDs in one Zimbabwean central hospital setting. Data was collected from participants who consented and was analyzed using Stata version 13. A four-point Likert scale was used to categorize different levels of perceived satisfaction.

**Findings:**

Twenty-three doctors participated in the study: four female doctors and nineteen males. Nineteen of the doctors were general practitioners, whilst four were specialists. The findings indicated that both categories perceived some shortfalls in clinical care for NCDs. Moreover, the perceptions of general practitioners and specialists were not significantly different. Participants perceived cancer care to be lagging far behind the other three NCDs under study. Care of cardiovascular diseases (CVDs) and diabetes showed mixed perceptions amongst participants, with positive perceptions almost equaling negative perceptions. Furthermore, hypertension was perceived to be clinically cared for better than the other NCDs under consideration. Reasons for the gaps in NCD clinical care were attributed by 33% of the participants to financial challenges; a further 27% to patient behavioral challenges; and 21% to communication challenges.

**Conclusions:**

The article concludes that care delivery for the selected NCDs under study at CCH need to be improved. Furthermore, it is crucial to diagnose NCDs before patients show clinical symptoms. This helps disease prognosis to yield better care results. The evaluation of doctors’ perceptions indicates the need to improve NCD care at the institution in order to control NCD co-morbidities that may increase mortality.

**Supplementary Information:**

The online version contains supplementary material available at 10.1186/s12992-021-00730-3.

## Introduction

Non-communicable diseases (NCDs) have recently become a global public health burden, contributing more than half of the global health loss [1]. NCDs account for between 60 and 70% of all deaths globally [1, 2]. NCDs are also a leading cause of premature death, 80% of which are in low- and middle-income countries (LMICs) [1]. The World Health Organization (WHO) also reported a 71% increase in morbidity and mortality related to NCDs, with more than 40.5 million deaths from the total 56.9 million global deaths recorded in 2016 being related to NCDs, three quarters of which were in LMICs [3, 4]. The NCD burden is exacerbated in LMICs due to co-morbidities with the HIV burden [5]. The WHO estimated that by 2020, NCDs were expected to result in approximately 80% of the worldwide disease burden, causing about seven in every ten deaths in LMICs [3, 6].

Apart from the high mortality and morbidity of NCDs, healthcare systems in most LMICs are generally ill-prepared, fragile, under-resourced and face infrastructural limitations in dealing with the epidemiological and economic costs associated with NCDs [6–8]. This is evident through disparities in health statuses and life-expectancies between high-income countries and LMICs, particularly in relation to NCDs, which is attributable to poor healthcare delivery and management systems in LMICs [9]. A healthcare delivery system is defined as a combination of organized people, organizations and resources for the purposes of delivering healthcare services to meet the health needs of a target population [10]. Healthcare services may be delivered by single-provider practices or a big healthcare ecosystem. Moreover, healthcare systems must provide quality healthcare services, whereby the World Health Organization defines “quality as the degree to which health services for individuals and populations increase the likelihood of desired health outcomes and are consistent with current professional knowledge” [11]. Hence, the quality of healthcare services affects the level of satisfaction, which refers to the fulfilment of physicians’ expectations in the healthcare delivery system for NCD patients.

It is apparent that LMICs need to improve the performance of their healthcare systems with regard to NCDs. Global efforts for the prevention and control of NCDs have intensified after the endorsement of the Global Strategy for the Prevention and Control of Non-communicable Diseases [12]. However, the effective prevention, control and management of NCDs requires properly planned healthcare systems and response mechanisms [11]. These response mechanisms must incorporate a multiplicity of stakeholders/sectors, including those not directly involved in health [13].

Literature records that NCDs are a major public health burden in the Sub-Saharan Africa (SSA) region, with significant morbidity and mortality [14]. A few studies have been conducted in Zimbabwe to assess the disease burden of selected NCDs, but the authors did not find literature evaluating the management and control of NCDs. For instance, Smit et al. (2015) examined the burden of NCDs in people living with HIV (PLHIV) in Zimbabwe [15]. The results showed that PLHIV had a 19.6% chance of being diagnosed with at least one NCD and a 4.6% likelihood of being diagnosed with more than one NCD [11]. Other studies focused on the prevalence of hypertension. Chimberengwa and Naidoo (2019) examined the knowledge, attitudes and practices related to hypertension in a rural setting in Zimbabwe [16]. Additionally, Chireshe and Naidoo (2019) studied hypertension amongst patients treated at Parirenyatwa Hospital, Zimbabwe’s biggest referral hospital, and found an incidence of hypertension of 29.9% [17]. In a meta-analysis, Mutowo et al. (2015) found a hypertension prevalence of 30%, with a higher burden in urban areas than in rural areas [18]. Overall, the WHO in Zimbabwe estimates that NCDs accounted for about 33% of the mortalities recorded in 2016 [19].

The management of NCDs in LMICs faces a myriad challenges such as poor laboratory facilities; frequent medicine stock-outs; a limited and poorly distributed health workforce and pharmaceuticals; and poor access to financial resources for caregivers and clients, amongst many other challenges [20, 21]. The challenges highlighted above also affect the management and control of NCDs in Zimbabwe.

The improvement of the management and control of NCDs by the Ministry of Health and Child Care (MoHCC) in Zimbabwe, in cooperation with the WHO, has been a strategic priority since 2016 [22]. The management of NCDs in Zimbabwe includes several personnel who are either directly or indirectly involved in the daily management of different NCDs. These personnel include physicians, laboratory scientists, nurses, pharmacists, administrators and community health workers, amongst others. Physicians are amongst the primary direct caregivers with the responsibility for screening, diagnosis, ordering further examinations, reviewing and treatment of NCD patients. The authors elected to evaluate physicians’ perceptions in order to gather expert opinions in light of the fact that physicians play a key role in healthcare, since the ultimate responsibility for patients’ care rests on them. This study can be a foundation for expanding the scope of research to evaluate perceptions by other NCD-care personnel.

The aim was to conduct an exploratory study to evaluate the availability and quality of NCDs clinical care based on the perceptions of physicians who attend to NCD patients at Chitungwiza Central Hospital (CCH), a referral hospital located in the Harare metropolitan province of Zimbabwe. The study was also motivated by the existence of scant literature on evaluations of care delivery systems for the management and control of NCDs by caregivers such as physicians in developing countries, including Zimbabwe.

## Materials and methods

### Study design

This was a qualitative exploratory study meant to obtain expert perceptions of care delivery for NCDs in one Zimbabwean referral hospital setting. Data was collected from participants who consented and was analyzed using Stata version 13.

### Study setting

The study was conducted at CCH, which is a referral hospital with a catchment area including urban, peri-urban and rural locations. There are about 15 hospitals in Mashonaland East Province surrounding CCH. In addition, CCH serves an estimated population of 1.5 million people, spread over an estimated 32,230 km^2^ area. CCH attends to an average of 80 NCD patients in the outpatients’ department and 20 in the inpatients’ department daily. The economic status of the population within the catchment area of the CCH is generally poor, with the majority living below the national poverty line, averaging US$220 monthly for a family of five [23], a common feature for most settings in Zimbabwe due to high unemployment levels. The population is therefore mostly unemployed or self-employed, embarking on livelihood sustenance projects such as market gardening, vending and other informal economic activities.

### Recruitment of participants

From a total population of 43 medical doctors engaged by CCH on a part-time or full-time basis, 26 doctors, including specialists, were recruited to participate in the study based on the inclusion/exclusion criteria provided below. Only doctors who consented to the study were included in the study, while doctors who do not provide clinical care for NCD-related conditions were excluded from participating in the study, For, example dental surgeons.

i) **Inclusion Criteria**

- Medical doctors employed by the CCH, both on a full-time or part-time basis, who provide clinical care to patients with NCD-related conditions.
- Only doctors who consented to participate in the study were included.

ii) **Exclusion Criteria**

- Other hospital staff who are not medical doctors.

### Data collection procedures

Data was collected from both sessional and resident doctors at CCH through a self-administered questionnaire. To ensure that there was minimum bias regarding the respondents’ perceptions, the anonymity of the participants was assured in both data collection and analysis. The questionnaires were distributed electronically through the Survey Monkey platform. The participants included general practitioners and specialists providing clinical care for NCD-related conditions.

The selection of study participants was based on their knowledge and experience in the management of NCDs. Therefore, doctors who do not provide clinical care for NCDs or any related conditions were excluded from the study. All the participants who met the inclusion criteria were communicated with in order to obtain their consent and thereafter, a link to the online questionnaire was distributed via email to the 26 participants who consented.

In order to assess the quality of clinical care, the World Health Organization’s definition of quality was used as the basis for assessing quality, that is, “the extent to which the healthcare services provided to individuals and patient populations improve desired health outcomes. To achieve this, healthcare must be safe, effective, timely, efficient, equitable and patient-centered” [10]. The quality of healthcare is a collaborative effort involving the patient, physician, patient’s family and the community. Therefore, it can be assessed from the perspectives of any of these. Accordingly, physicians’ perceptions of quality were assessed based on the features highlighted in the above definition, measured using a four-point Likert scale with the following options:

**Table:** 

Not satisfactory	Somehow satisfactory	Satisfactory	Very satisfactory
1	2	3	4

The Likert scale was used to quantify the qualitative evaluations of physicians’ perceptions of clinical care with the following meanings: *Not satisfactory* means that physicians perceived the clinical care to be unable to meet the healthcare service expectations; *Somehow satisfactory* means that physicians were indifferent about the NCD clinical care, that is, it was meeting doctors’ expectations to a lesser extent; *Satisfactory* means that the physicians perceived the clinical care to be just meeting basic healthcare needs; and *Very satisfactory* means that the physicians perceived the clinical care to be world-class and going beyond expectations.

### Data analysis procedures

Data was analysed using Thematic Analysis [24]. Since some of the questions were open-ended, open coding was done, capturing the frequency of the major themes which are presented in tables and bar graphs. Stata version 13 was used for the statistical analysis of data and to plot bar graphs. Qualitative data was open coded first and subsequently axial coded before presentation and analysis [22]. The analysis of data was guided by themes that emerged from the data and related responses were analyzed together [22].

### Ethical consideration

Ethical approvals for the study were received from the Biomedical Research Ethics Committee of the University of KwaZulu-Natal (**BE057/19**) and the Medical Research Council of Zimbabwe (**MRCZ/A/2441**). Participants provided informed consent prior to data collection.

## Results

Twenty-six participants consented to the study, 23 of which completed and uploaded their responses. These are valid for the study, giving a response rate of 88.5%. Their demographic characteristics are presented in Table 1 below.

**Table 1:** Demographic Characteristics of Respondents (*n* = 23)

Features	Description	Frequency	Percentage (%)
Gender	Male	19	82.6
	Female	4	17.4
Doctors’ specialization	Specialist	4	17.4
	General practitioner	19	82.6

**Fig. 1:**
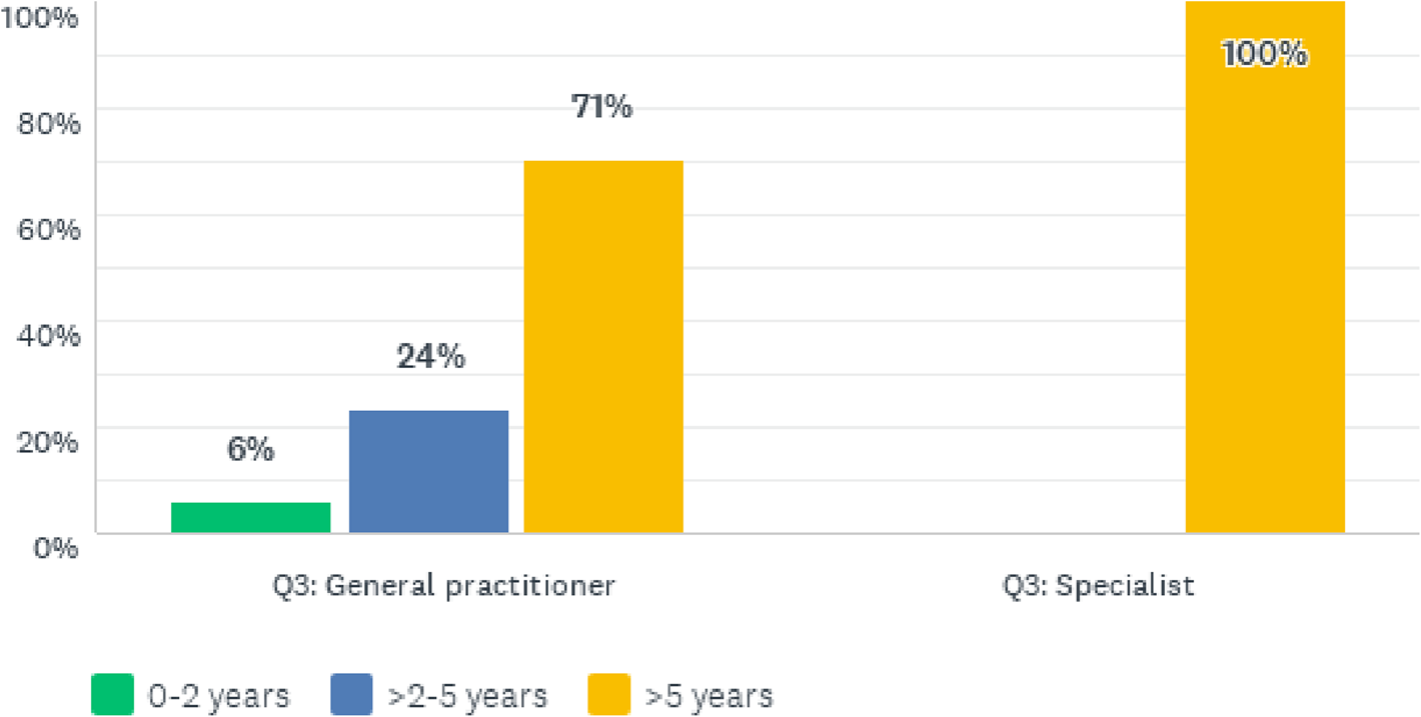
Participants’ experience offering clinical care to NCD patients

As shown in Tables 1, 17.4% of the valid responses were from female doctors, whilst 82.6% were from male doctors. Although most of the participants were general practitioners (82.6%), the doctors were well experienced in providing clinical care for NCDs, as shown in Fig. 1.

**Fig. 2:**
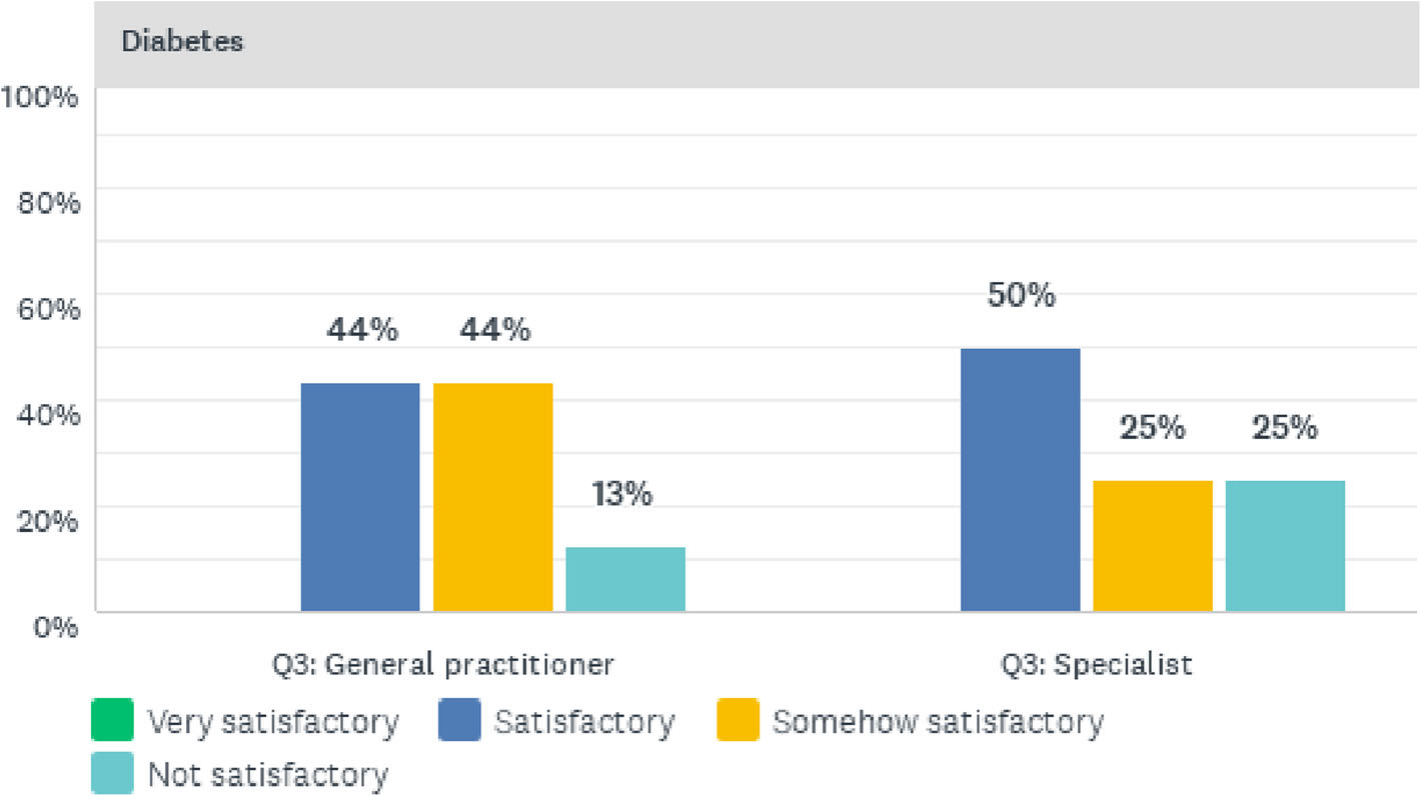
Quality of diabetes clinical care services offered at CCH

As shown in Fig. 1, all specialists had been offering clinical care to NCD patients for more than 5 years, whilst 71% of general practitioners had more than 5 years of clinical care experience for NCD patients. The doctors gave their perceptions of the quality of clinical care services rendered to NCD patients. These responses are presented hereunder, starting with Fig. 2 depicting the physicians’ perceptions of diabetes care at CCH.

Figure 2 represents doctors’ perceptions of the quality of clinical care services for diabetes. The quality of clinical care services for diabetic patients at CCH was perceived by 44% of general practitioner doctors as being satisfactory, whilst a further 44% of the general practitioners viewed the quality as somewhat satisfactory. The remaining 12% regarded diabetes clinical care to be unsatisfactory. In addition, 50% of the specialists regarded clinical care to be satisfactory and 25% regarded diabetes clinical care to be somewhat satisfactory. However, 25% of the specialists perceived the clinical care services for diabetic patients as not satisfactory.

**Fig. 3:**
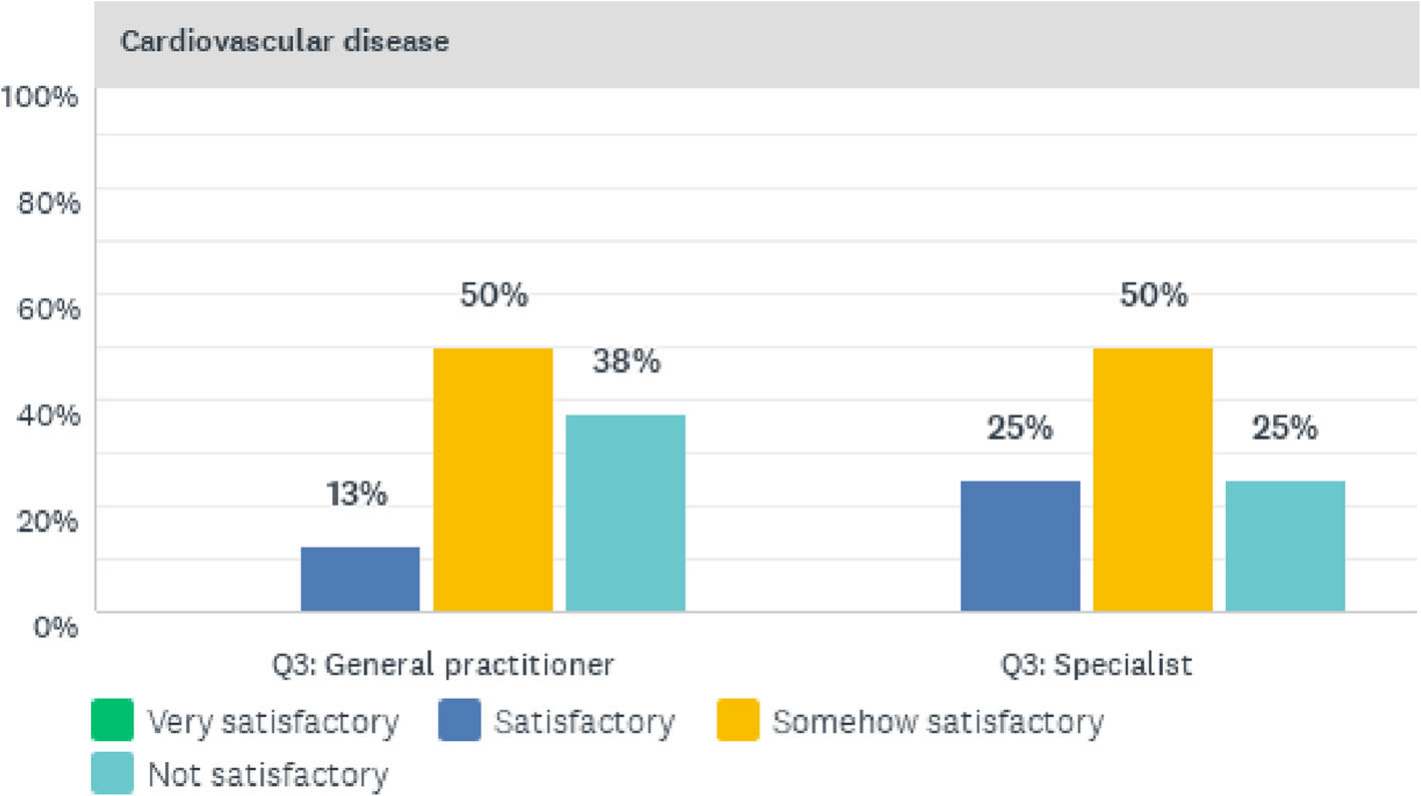
Quality of CVD clinical care services offered at CCH

Figure 3 below presents a summary of the physicians’ perceptions of clinical care for cardiovascular diseases (CVD).

The quality of clinical care services rendered to CVD patients was perceived by 50% of specialists and 50% of general practitioners as being somewhat satisfactory. A further 12% of general practitioners and 25% of specialists regarded the quality of clinical care to be satisfactory, whilst 38% of general practitioners and 25% of specialists viewed the quality of CVD clinical care as not satisfactory.

**Fig. 4:**
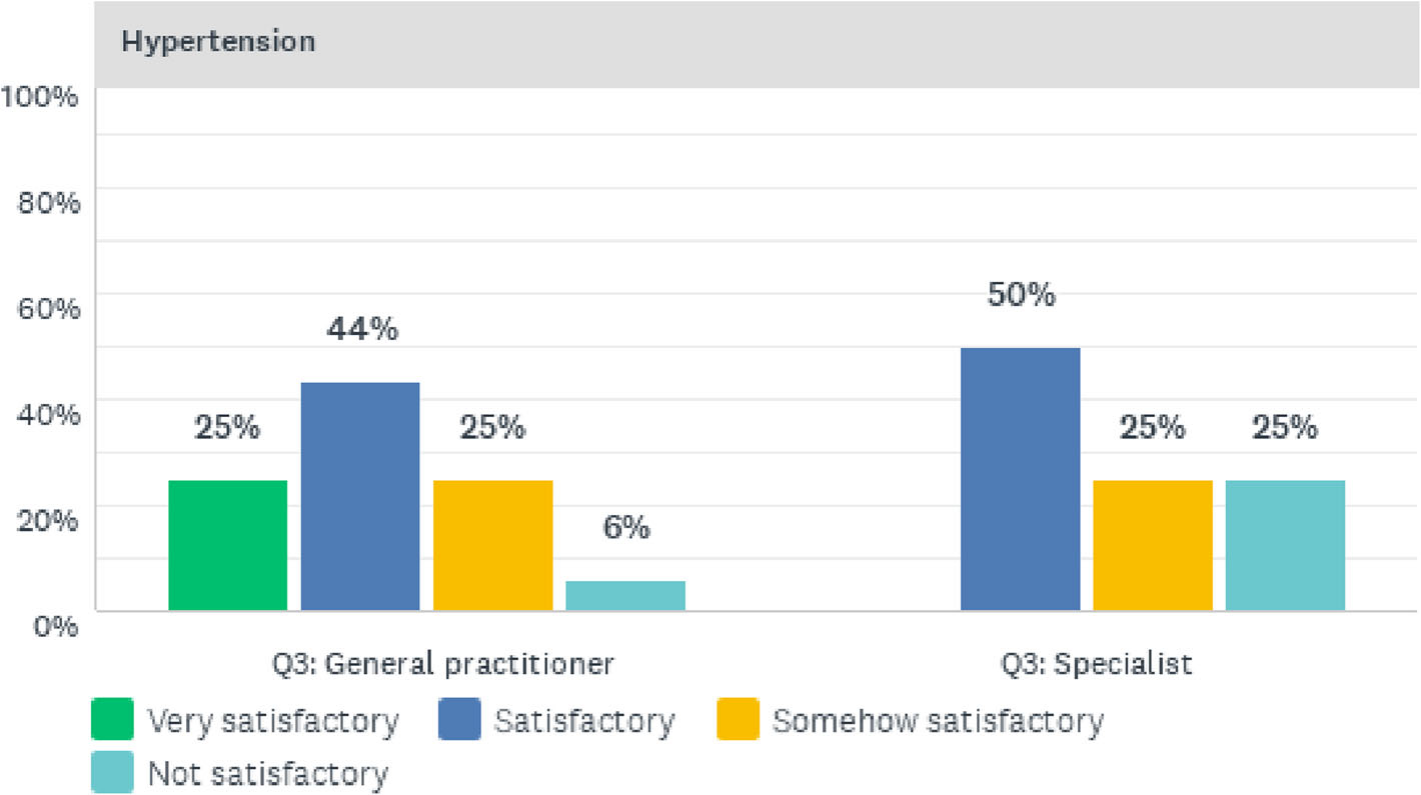
Quality of hypertension clinical care services offered at CCH

The physicians’ perceptions of the quality of clinical care for the management of hypertension showed that 25% of general practitioners regarded the quality of clinical care services as very satisfactory. In addition, 50% of specialists and 44% of general practitioners perceived the quality of clinical care for hypertension to be satisfactory. Furthermore, 25% of both specialists and general practitioners regarded the quality of clinical care to be somewhat satisfactory; whilst 25% of specialists and 6% of general practitioners regarded the quality of clinical care to be not satisfactory. These results are shown in Fig. 4.

**Fig. 5:**
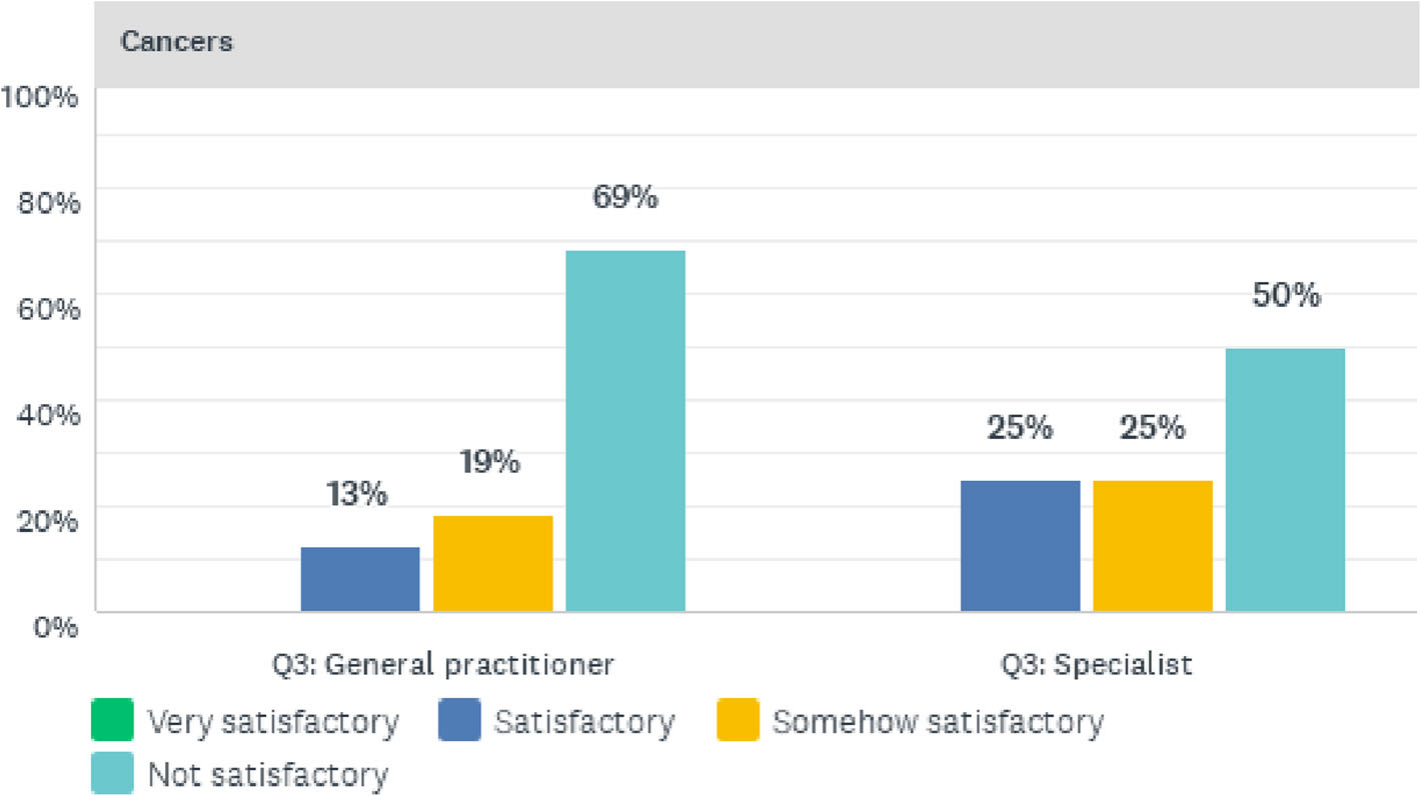
Quality of cancer clinical care services offered at CCH

Lastly, in relation to the management of cancers, the doctors evaluated the quality of clinical care and the results of their perceptions are presented in Fig. 5. A majority of both general practitioners (69%) and specialists (50%) indicated that the quality of clinical care was not satisfactory. In addition, 25% of specialists and 19% of general practitioners perceived the quality of care as somewhat satisfactory, whilst a further 25% of specialists and 13% of general practitioners regarded the quality of cancer clinical care as satisfactory.

**Fig. 6:**
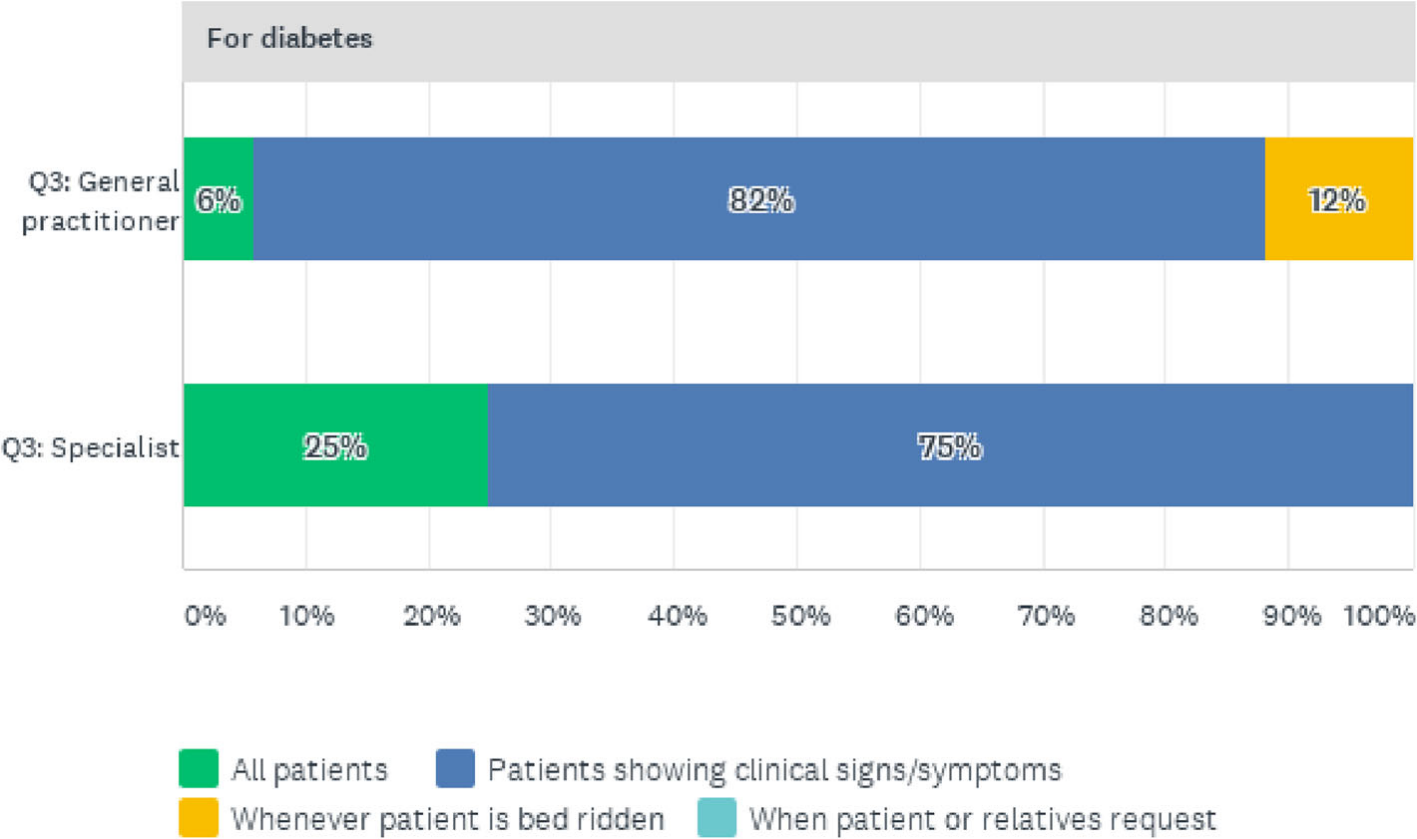
Reasons for requesting Diabetes Screening Tests for Patients

Given the perceptions of both general practitioners and specialist doctors of the quality of clinical care, it was important to ascertain the circumstances under which these physicians would request further tests and diagnosis. Screening and testing are critical for the effective management of NCDs since early detection makes most NCDs manageable and less fatal. The results are presented separately for the four NCDs under study. Figure 6 presents the reasons for both specialist and general practitioner doctors requesting testing for diabetic patients. It is observed that it is common practice for general practitioners (82%) and specialists (75%) to request further diabetes tests for patients presenting clinical symptoms. The rest of the reasons are shown in Fig. 6.

**Fig. 7:**
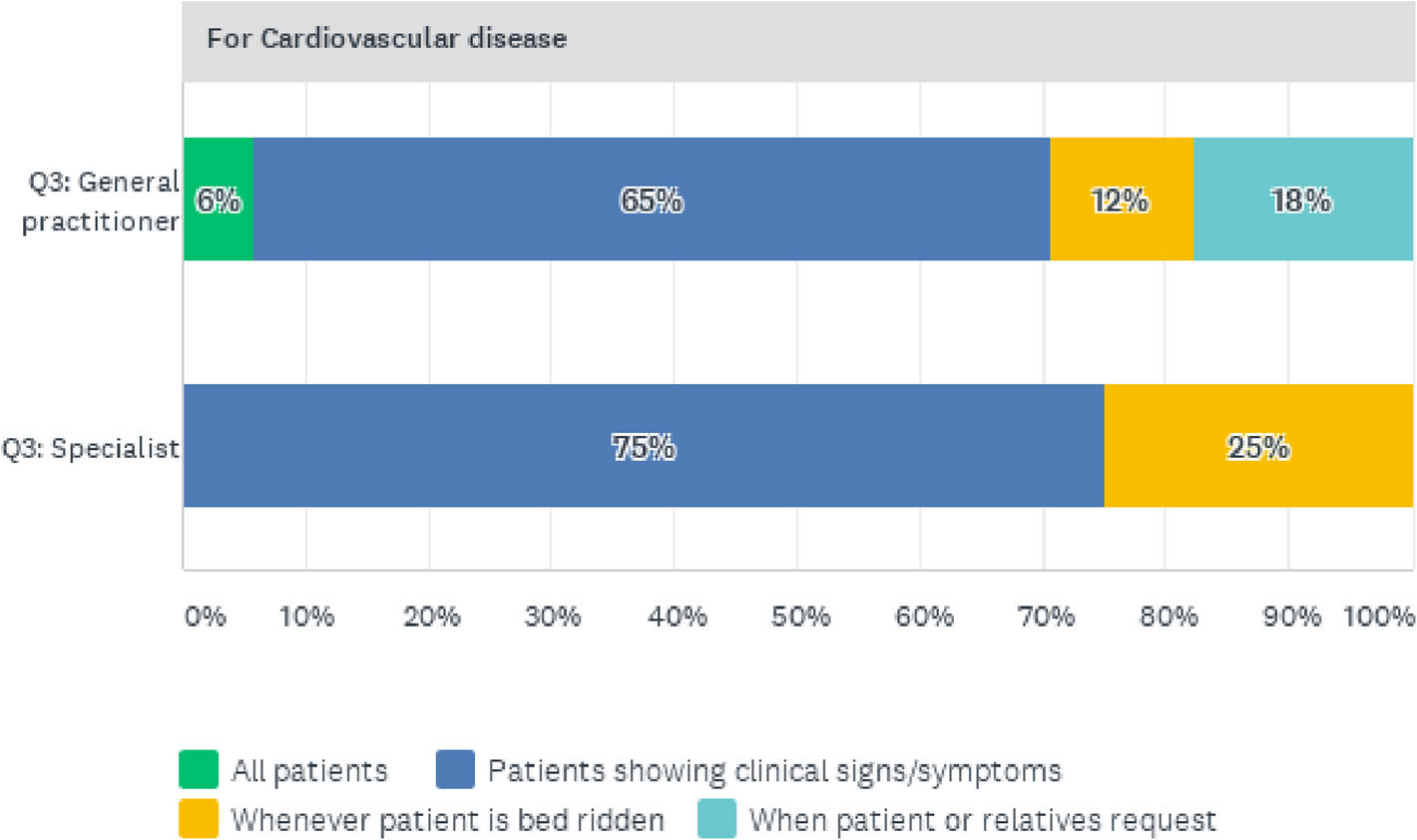
Reasons for requesting CVD Screening tests for patients

As presented in Fig. 7, it was also common practice to request screening tests for CVD patients presenting clinical signs and symptoms, as shown by 75% of specialists and 65% of general practitioners.

**Fig. 8:**
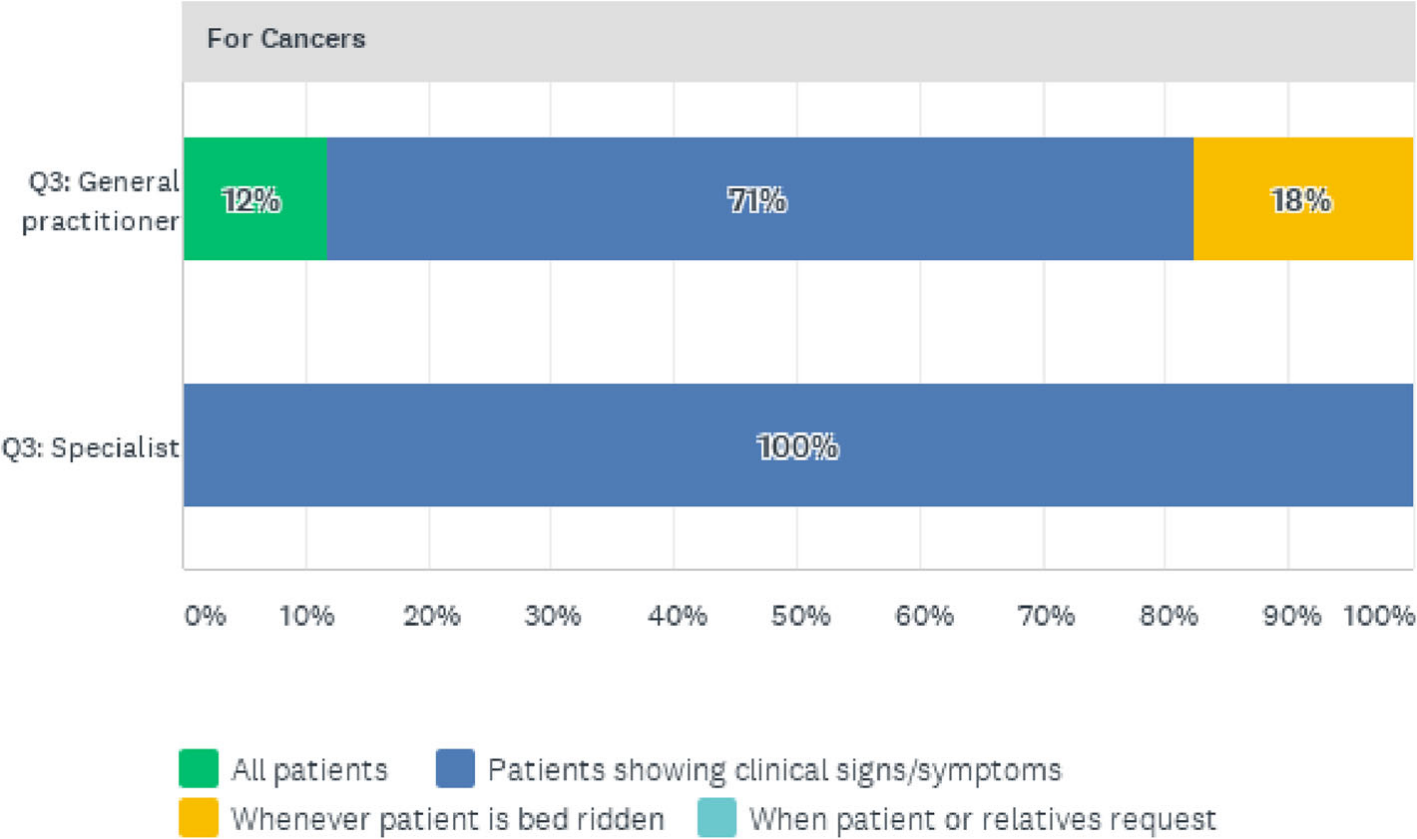
Reasons for requesting Cancer Screening Tests for Patients

The predominant reasons for screening and testing for cancers, as shown in Fig. 8, are similar to CVDs and diabetes, as both specialists (100%) and general practitioners (71%) mainly requested screening for patients presenting with clinical signs and symptoms.

**Fig. 9:**
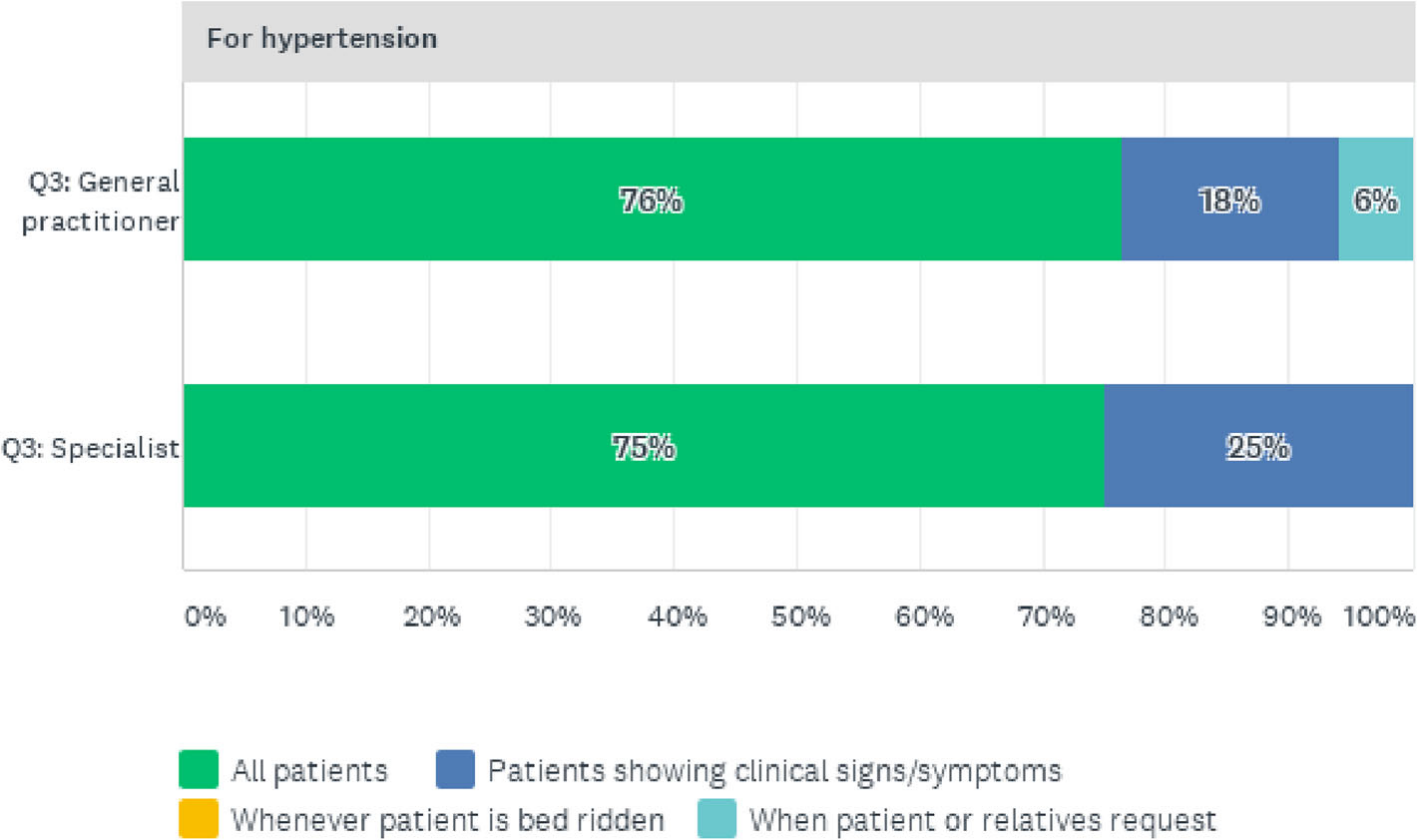
Reasons for Hypertension Screening Tests for Patients

Of the four NCDs under study, hypertension presented a dissimilar predominant reason for screening and testing whereby almost all patients had their blood pressure checked when being attended to as part of the admission procedure. The reasons for testing for hypertension are shown in Fig. 9.

After establishing the conditions under which doctors requested further screening tests for effective management, the challenges that doctors faced when providing clinical care to NCD patients were enquired from the doctors in order to establish their perspectives. The responses obtained in relation to these challenges are grouped by themes into related challenges and are summarized in Table 2 below.

**Table 2:** Challenges faced by Doctors providing quality NCD clinical care at CCH

**Financial Challenges**	**Patient Behavioral Challenges**
No money for laboratory tests.	Non-compliance with lifestyle changes and denial.
Unavailability of affordable screening services.	Non-compliance with treatment.
Drugs are unaffordable.	Religious and Traditional beliefs which cause bad health-seeking behavior.
Lack of funding from government.	Lack of co-operation from patients.
**Supply Chain Challenges**	**Communication Challenges**
Shortages of screening tools and equipment for tests.	No clear guidelines for NCD patients.
Unavailability of drugs.	Poor information dissemination.
Shortages of advanced equipment.	A lack of basic knowledge about risk factors by the populace.
**Care provider challenges**	
No special testing laboratories.	
Shortages of specialists.	
Lack of expertise to support the patients.	

**Fig. 10:**
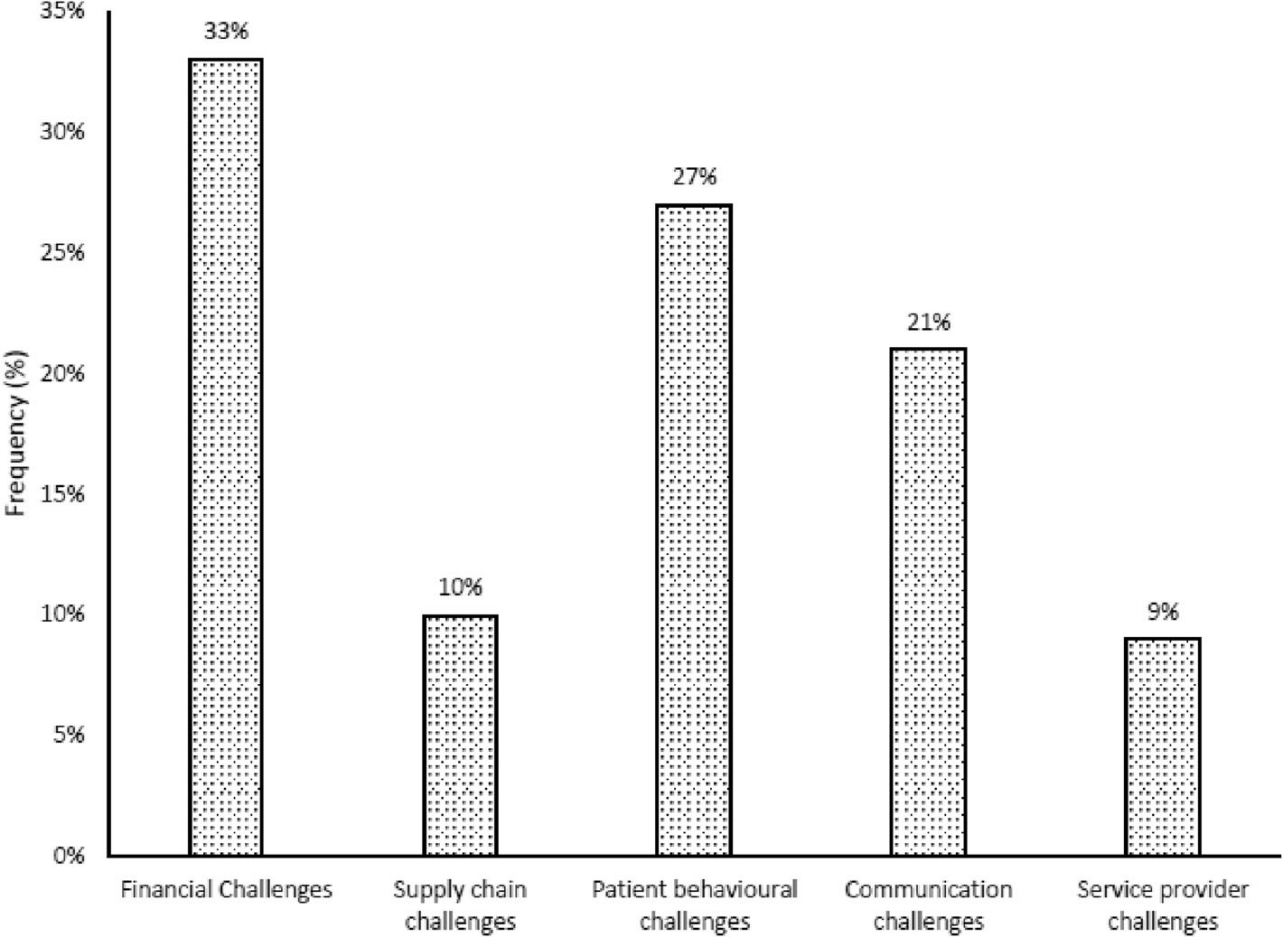
Categorized Challenges Faced by Doctors Caring for NCDs at CCH

Table 2 shows the five categories of challenges identified by the study, namely financial challenges, patient behavior challenges, supply chain challenges, communication challenges, as well as care provider challenges. The statistics regarding the highlighted challenges are provided in Fig. 10.

The most cited challenges were financial challenges, as perceived by 33% of the doctors. This was followed by patient behavioral challenges, which was cited by 27% of the doctors at CCH. Another 21% of the doctors viewed communication as a challenge in the provision of care for NCD patients, whilst a further 10% cited challenges in the medical supply chain and 9% cited challenges with care providers for NCD care.

**Fig. 11:**
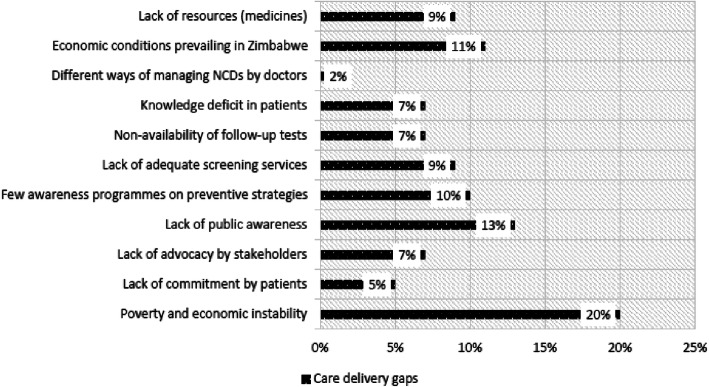
Care Delivery Gaps

Given the challenges presented in Table 2 and summarized in Fig. 10, the study enquired about the gaps that the doctors considered to be stumbling blocks in the provision of care for NCD patients. The gaps established are summarized in Fig. 11 below.

The gap cited by the highest number of respondents was poverty and economic instability, as 20% of the doctors identified this factor as affecting the delivery of care to patients. The least identified gap was the varied methods used by doctors to manage different NCD patients, as cited by only 2% of the doctors. This shows a need for a standard framework for the screening and management of NCDs. Possible mitigatory measures which could be implemented to improve the situation are summarized in Table 3 below.

**Table 3:** Mitigatory Measures to Close the Care Delivery Gaps for NCDs Patients

Possible mitigatory measures that could be implemented	Percentage (%)
Mandatory hospital-based tutoring for the public	7
Educational awareness campaigns	14
Better information dissemination through the media	7
A policy that speaks to NCDs	7
Free treatment at designated hospitals	10
Integration of health provision	6
Decentralization of NCD clinics to district hospitals	4
Screening tests at each clinic visit	5
Availing of funding from central government and development partners	12
Set up specific NCD clinics and help support them by providing adequate stocks of medication and laboratory equipment support	3
Affordable health insurance schemes	10
Give flexible timetables for patients’ visits	7
Procure medicines on time	7

As shown in Table 3, several possible measures are highlighted which could be implemented at CCH in order to mitigate the extent of NCDs and improve care delivery for NCD patients. According to 14% of the doctors, educational campaigns could help in mitigating the exposure of patients to various NCDs. A further 12% viewed the provision of funding from central government and development agencies as an effective strategy to mitigate the challenges faced in providing effective care to NCD patients. The strategies presented in Table 3 would be helpful in dealing with NCDs at public health institutions in Zimbabwe. The effectiveness of these strategies was not covered by this study and may require further enquiry.

## Discussion

The aim of the study was to evaluate the availability and quality of NCDs clinical care based on the perceptions of physicians who attend to NCD patients at Chitungwiza Central Hospital (CCH), a referral hospital located in the Harare metropolitan province of Zimbabwe. The findings indicated perceived gaps in care delivery for NCD patients attended to at CCH. The authors found that a significant number of the doctors thus viewed the quality of the care services offered to NCD patients to be unsatisfactory overall. This was attributable to various challenges impeding the smooth flow of care delivery for the selected NCDs.

The study also assessed the screening procedures for diabetes, hypertension, CVD and cancers. The authors observed that except for hypertension, which was screened for the majority of patients, the doctors first checked clinical signs and symptoms in order to request further tests. This is a matter of concern since NCDs may take time to be evident clinically, and in most cases will show signs at advanced stages, leading to poor prognosis of the condition. Devi, et al. elaborate further on the necessity of including screening and diagnosis of NCDs in the models for NCD management [8]. The study also identified the challenges faced by doctors in the management of NCDs. The most prevalent challenges were financial where the patients could not afford some of the procedures and services ordered by the physicians to manage the condition for better prognosis. The least challenging obstacle related to service provider challenges, since the study setting was in an urban location which is not badly affected by human resources challenges, which are common impediments in rural areas [25]. However, the researchers could not rule out possible bias since the participants were caregivers.

Doctors who participated in the study identified several gaps in clinical care delivery for NCD patients at CCH and proposed strategies that could be pursued in order to improve the quality of clinical care. Some of the proposed strategies include pursuing hospital-based tutoring to educate the community on the risk factors, disease burden and mortality associated with NCDs. CCH management and the responsible ministry should consider providing mandatory and free testing for NCDs for all patients in order to ensure early detection. This enhances the quality and effectiveness of the care and management of NCDs. Medical institutions such as CCH can improve clinical care quality through availing resources, medication and equipment for NCD screening and management at primary healthcare institutions.

The doctors’ perceived unsatisfactory quality of clinical care delivery for NCD patients was consistent with findings obtained from other studies in almost similar settings, especially those in the SSA region [2, 6, 7]. Poverty has been cited as a common cause for high NCD prevalence and the unsatisfactory care of NCD patients, as well as for the high mortality rates for various NCDs in the SSA region and LMICs [3, 5, 8]. This is attributable to various reasons, which include incapacitation to meet hospital bills for general medical check-ups, which are key to the early diagnosis of NCDs [5, 8). Moreover, poverty compromises educational achievements, which impedes the knowledge base on prevention and the lifestyle changes necessary to minimize exposure to NCDs [3, 25). The chronic nature of NCDs makes most of the populations from LMICs unable to afford the lifelong treatment required because of poverty [2].

Challenges faced in the care delivery for NCD patients were not unique to doctors at CCH. Due to economic challenges in developing countries, financial challenges are common for NCD care providers in developing countries and the SSA region [2, 7, 8]. Doctors also highlighted perceived challenges related to patients’ behavior, which exposes them to higher NCD risks [4]. Behaviors identified in literature include absconding from treatment due to stigma or the inability to afford medication, as well as religious practices such as faith-healing beliefs resulting in medication abscondment [4, 5, 8]. The study also revealed doctors’ perceptions of the existence of behaviors by patients, which compromise care delivery for NCDs [6, 10]. Patient behaviors and attitudes are common challenges in medicine whereby adherence to prescriptions is neglected and patients are sometimes unwilling to change behaviors such as smoking and alcohol abuse, despite the resultant exposure to undesirable effects such as NCD development [4, 5].

The strength of this exploratory study was primarily its contribution to an initial evaluation of NCD patients’ care delivery from a referral hospital in a developing country setting. The study population comprised medical doctors who are experts with varied experience in the management of NCDs. The study thus contributed towards the body of knowledge on the management of NCDs from an expert viewpoint. This research presents a baseline study that can guide further studies on evaluating NCD care delivery using either an expanded population from CCH or incorporating other care-givers in order to ascertain gaps and strategies for enhancing care delivery.

However, the main limitation of the study was the limited geographical coverage and the small sample used, which meant that the findings obtained in the study were not generalizable to be representative of the entire country and its health professionals. Moreover, there is a possibility of bias by respondents since the study was a self-evaluation. Therefore, doctors were unlikely to highlight their own weaknesses in NCD clinical care provision. The exclusion of patient observations was another limitation of the study. However, these limitations cannot invalidate the findings, since the study was exploratory in nature and hence there is need for conducting a study with a bigger population and sample.

The authors recommend that the management at CCH consider a deliberate policy for enhancing the quality of clinical care provided to NCD patients through the development and implementation of a service level agreement. Since CCH is a provincial hospital, the proposed policy can be disseminated to district clinics and hospitals. It is also recommended that all medical staff such as nurses be capacitated to offer acceptable quality NCD care to patients. Moreover, clinical processes must be re-engineered to ensure that NCD care is an integral part of patient care procedures at the institution. These recommendations are envisaged as having the ability to improve the quality of NCD clinical care at CCH.

## Conclusions

The study’s findings showed that the clinical care offered for most NCDs at CCH is not of the quality and standard expected by the physicians to reduce morbidity and mortality. The quality of clinical care is compromised by challenges affecting healthcare institutions such as CCH. There is an unavailability of sufficient equipment and/or affordable services for NCD care. Medical supplies are also limited in terms of availability and affordability. In addition, physicians at CCH perceived the existence of informational and knowledge gaps in the care and management of NCDs by patients. Doctors also observed that NCD patients fail to adhere to treatment protocols due to religious and traditional beliefs.

Based on the findings obtained in the study, the following conclusions were drawn regarding doctors’ perceptions of care delivery for NCD patients at CCH:

- Care delivery for NCD patients is perceived by some doctors as being unsatisfactory in meeting the requirements of the patients since there were challenges impeding their delivery of clinical care to NCD patients.
- There are no clear guidelines and policies for healthcare practitioners on clinical care delivery for NCD patients. As a result, doctors do not have benchmarks for care delivery, as was evidenced by the varied responses from the doctors regarding the care of patients.
- There are no standard protocols for screening and managing NCDs, which encourages the early diagnosis of NCDs. This is likely to improve the quality of care, resulting in care which is safe, effective, timely, efficient, equitable and patient-centered.

The study is important in giving direction to both doctors and other stakeholders at CCH and in Zimbabwe on areas of possible improvement in NCD care delivery. The evaluation of doctors’ perceptions of NCD clinical care delivery at CCH enlightens hospital management and policy-makers on the need to improve NCD care at the institution in order to control NCD co-morbidities that may increase mortality. Findings from the study can also inform other low-income countries facing an escalation of NCDs burden and need to improve their clinical care delivery for persons diagnosed with NCDs. The study showed a need to improve timely NCD diagnosis, and the earlier commencement of care will ultimately give better results for the patient management. Policy makers, hospital management, and clinical caregivers in comparable settings can draw valuable lessons on ways to improve NCDs management and customize the findings to their unique clinical care settings.

## Supplementary Information

**Additional file 1. **Questionnaire

**Additional file 2.** Anonymized Study Dataset.

## Data Availability

The data that support the findings of this study are available and an anonymized dataset is included as an additional supporting file.

## Abbreviations

AIDS: Acquired Immune Deficiency Syndrome
CCH: Chitungwiza Central Hospital
CVD: Cardiovascular disease
HIV: Human Immunodeficiency Virus
LMICs: Low to Medium Income Countries
NCDs: Non-Communicable Diseases
OI: Opportunistic Infections
PLHIV: People Living with Human Immunodeficiency Virus
SSA: Sub-Saharan Africa
