# Supplementary material for: A qualitative exploratory study of selected physicians’ perceptions of the management of non-communicable diseases at a referral hospital in Zimbabwe
Source: Global Health. 2021 Jul 19;17:82. doi: 10.1186/s12992-021-00730-3 (PMC8287754; doi:10.1186/s12992-021-00730-3)
Supplement: Supplementary file 1 — Additional file 1. Questionnaire [file 12992_2021_730_MOESM1_ESM.docx]

Q1: Gender

Q2: How long have you been attending to patients suffering from NCDS (Non-communicable diseases)


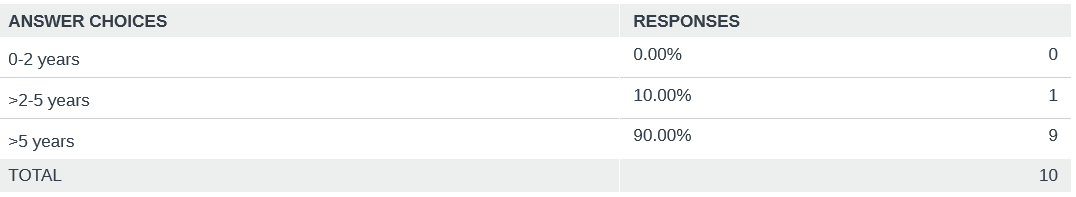


Q3: What stage are you in the field?

Q4: How do you rate the services being rendered to patients suffering from the following conditions in the table below at Chitungwiza Central Hospital. [Please put a tick under your choice]

Q5: What usually prompts you to request a test to screen or diagnose each of the conditions? You can choose more than one reason for each.

Q6: What challenges do you face with people suffering from noncommunicable diseases?

What challenges do you face with people suffering from noncommunicable diseases?

| Challenges 1 | Challenges 2 |
| --- | --- |
| No clear guidelines for Zimbabwe patients | Lack of availability of newer better drugs |
| Noncompliance with lifestyle changes | Unavailability of drugs |
| Financial constraints | Poor monitoring |
| Defaulting medications | Traditional belief which cause bad health seeking behaviour |
| Shortage of screening tools | Unavailable medication |
| Less screening | Unavailability of affordable screening services |
| Expensive medications | Drugs are expensive so default treatment. |
| Non-compliance to treatment. | Lack of resources |
| Non availability of diagnostic | No drugs |
| Lack of cooperation with patients |  |
| CHALLENGES 3 | **CHALLENGES 4** |
| No dietary guidelines for our local context | Shortage of specialists |
| Unavailability of equipment for tests | Late presentations |
| Lack of medications | Lack of awareness |
| Denial | Religion |
| No money for laboratory tests | No adequate manpower |
| No medications | Lack of expertise to support |
| Lack of government support |  |
| CHALLENGES 5 | **CHALLENGES 6** |
| Poor information dissemination | A lack of basic knowledge by the populace |
| Lack of investigations for follow ups | Lack of communication |
| Poor knowledge on condition. | Lack of funding from govt |
| No food for them |  |
| No special testing at laboratories |  |

Q7: In your opinion, what are the gaps hindering maximum care delivery for NCDs?

| Poverty and economic instability | Expensive treatment options |
| --- | --- |
| Lack of commitment by patients | Expensive cancer drugs |
| Lack of advocacy by stakeholders | Lack of specialization |
| Lack of public awareness | Non compliance to medication |
| Few of awaress programs on preventive strategies | Religious beliefs |
| they are neglected | Lack of laboratory support in monitoring |
| Lack of awareness | Lack of awareness on NCDs in the community |
| Lack of adequate screening services | Expensive laboratory tests |
| Non availability of follow up tests | Lack of knowledge |
| Lack of medication | Lack of donor funding Competing health providers |
| Knowledge deficit in patients | High doctor to patient ratio |
| Different why of managing ncds by different doctors | Lack of medicine |
| Economic conditions | Crowded outpatients |
| Limited resources | Poor health policies with no clear and unmeasurable outcomes or expectations |
| Lack of medicines | Non availability of support staff |
| Lack of resources | Lack of medicines |

Q8: From the gaps identified above in (5), briefly state possible mitigatory measures that can be implemented.

| Mandatory Hospital based tutoring | Availability of drugs at health institutions |
| --- | --- |
| Educational awareness campaigns | Better policy formulation |
| Better information dissemination through media | Set up specific ncd clinics and help support them by stocking adequently and provide lab support |
| Health awareness campaigns | Source funds for treatment |
| Increase awareness programs in the community | Affordable Health Insurance Scheme |
| Teach the population | Free tests for Ncds |
| Policy that speaks to NCDs | Availing resources |
| Free drugs | Give flexible timetables |
| Training |  |
| Teach communities |  |
|  |  |
| Integration of health provision | Training specialist |
| Decentralization of NCD clinics to district hospitals | Supply of glucotests and insulin for free. |
| Screening tests on each clinic visit | Create a smooth flow of care |
| Workplace and Community Screening initiatives | Procure medicines in time |
| Free treatment at designated hospitals. | Decentralized care to all corners of the country |
| Apply for funding |  |
