# Supplementary material for: A qualitative exploratory study of selected physicians’ perceptions of the management of non-communicable diseases at a referral hospital in Zimbabwe
Source: Global Health. 2021 Jul 19;17:82. doi: 10.1186/s12992-021-00730-3 (PMC8287754; doi:10.1186/s12992-021-00730-3)
Supplement: Supplementary file 2 — Additional file 2. Anonymized Study Dataset. [file 12992_2021_730_MOESM2_ESM.pdf]

Anonymized Study Dataset

PID	Respondent ID	Collector ID	Gender	How long have you been attending to patients suffering from NCDS (Non-communicable diseases)	What stage are you in the field?	How do you rate the services being rendered to patients suffering from the following conditions in the table below at Chitungwiza Central Hospital. [Please put a tick under your choice]			
			Response	Response	Response	Diabetes	Cardiovascular disease	Hypertension	Cancers
1	12544143775	236815576	Male	>5 years	General practitioner	Satisfied	Not satisfied	Very satisfied	Not satisfied
2	12544113622	236815576	Male	>5 years	General practitioner	Somehow unsatisfied	Not satisfied	Satisfied	Not satisfied
3	12544029019	236815576	Male	>2-5 years	General practitioner	Satisfied	Not satisfied	Very satisfied	Not satisfied
4	12544010117	236815576	Male	>5 years	General practitioner	Somehow unsatisfied	Somehow unsatisfied	Satisfied	Not satisfied
5	12543877277	236815576	Female	>2-5 years	General practitioner	Satisfied	Not satisfied	Satisfied	Not satisfied
6	12543851712	236815576	Female	>5 years	General practitioner	Satisfied	Somehow unsatisfied	Satisfied	Somehow unsatisfied
7	12543753377	236815576	Male	>5 years	General practitioner	Somehow unsatisfied	Not satisfied	Satisfied	Not satisfied
8	12543682104	236815576	Male	0-2 years	General practitioner	Satisfied	Satisfied	Very satisfied	Satisfied
9	12542391758	236815576	Male	>2-5 years	General practitioner	Satisfied	Somehow unsatisfied	Very satisfied	Somehow unsatisfied
10	12542166298	236815576	Male	>5 years	Specialist	Satisfied	Satisfied	Satisfied	Somehow unsatisfied
11	12542152626	236815576	Male	>5 years	Specialist	Satisfied	Somehow unsatisfied	Satisfied	Not satisfied
12	11045742735	236815576	Female	>5 years	General practitioner	Not satisfied	Not satisfied	Not satisfied	Not satisfied
13	10946759234	236815576	Male	>5 years	General practitioner				
14	10945142064	236815576	Male	>5 years	Specialist	Not satisfied	Not satisfied	Not satisfied	Not satisfied
15	10942266125	236815576	Male	>5 years	General practitioner	Somehow unsatisfied	Somehow unsatisfied	Somehow unsatisfied	Not satisfied
16	10941662096	236815576	Male	>5 years	General practitioner	Somehow unsatisfied	Somehow unsatisfied	Somehow unsatisfied	Not satisfied
17	10940526824	236815576	Male	>5 years	Specialist	Somehow unsatisfied	Somehow unsatisfied	Somehow unsatisfied	Satisfied
18	10940266038	236815576	Female	>5 years	General practitioner	Somehow unsatisfied	Somehow unsatisfied	Somehow unsatisfied	Not satisfied
19	10939898917	236815576	Male	>5 years	General practitioner	Somehow unsatisfied	Somehow unsatisfied	Somehow unsatisfied	Somehow unsatisfied
20	10925246304	236815576	Male	>5 years	General practitioner	Not satisfied	Somehow unsatisfied	Satisfied	Not satisfied
21	10925212223	236815576	Male	>2-5 years	General practitioner	Satisfied	Satisfied	Satisfied	Satisfied
22	10783643240	236815576	Male			Satisfied	Somehow unsatisfied	Satisfied	Not satisfied
23	10783242843	236815576	Male			Very satisfied	Satisfied	Satisfied	Very satisfied

PID	Respondent ID	What usually prompts you to request a test to screen or diagnose each of the conditions? You can choose more than one reason for each.			
		For diabetes	For Cardiovascular disease	For hypertension	For Cancers
1	12544143775	Patients showing clinical signs/symptoms	Patients showing clinical signs/symptoms	All patients	Patients showing clinical signs/symptoms
2	12544113622	Patients showing clinical signs/symptoms	When patient or relatives request	When patient or relatives request	Patients showing clinical signs/symptoms
3	12544029019	Patients showing clinical signs/symptoms	Whenever patient is bed ridden	All patients	Patients showing clinical signs/symptoms
4	12544010117	Patients showing clinical signs/symptoms	When patient or relatives request	All patients	Whenever patient is bed ridden
5	12543877277	Whenever patient is bed ridden	Patients showing clinical signs/symptoms	All patients	Patients showing clinical signs/symptoms
6	12543851712	Patients showing clinical signs/symptoms	Patients showing clinical signs/symptoms	All patients	Patients showing clinical signs/symptoms
7	12543753377	Patients showing clinical signs/symptoms	Patients showing clinical signs/symptoms	Patients showing clinical signs/symptoms	Whenever patient is bed ridden
8	12543682104	Whenever patient is bed ridden	When patient or relatives request	Patients showing clinical signs/symptoms	Patients showing clinical signs/symptoms
9	12542391758	Patients showing clinical signs/symptoms	Whenever patient is bed ridden	All patients	Whenever patient is bed ridden
10	12542166298	Patients showing clinical signs/symptoms	Whenever patient is bed ridden	All patients	Patients showing clinical signs/symptoms
11	12542152626	Patients showing clinical signs/symptoms	Patients showing clinical signs/symptoms	All patients	Patients showing clinical signs/symptoms
12	11045742735	Patients showing clinical signs/symptoms	Patients showing clinical signs/symptoms	All patients	Patients showing clinical signs/symptoms
13	10946759234	Patients showing clinical signs/symptoms	Patients showing clinical signs/symptoms	All patients	Patients showing clinical signs/symptoms
14	10945142064	All patients	Patients showing clinical signs/symptoms	All patients	Patients showing clinical signs/symptoms
15	10942266125	Patients showing clinical signs/symptoms	Patients showing clinical signs/symptoms	All patients	Patients showing clinical signs/symptoms
16	10941662096	All patients	All patients	All patients	All patients
17	10940526824	Patients showing clinical signs/symptoms	Patients showing clinical signs/symptoms	Patients showing clinical signs/symptoms	Patients showing clinical signs/symptoms
18	10940266038	Patients showing clinical signs/symptoms	Patients showing clinical signs/symptoms	All patients	Patients showing clinical signs/symptoms
19	10939898917	Patients showing clinical signs/symptoms	Patients showing clinical signs/symptoms	All patients	All patients
20	10925246304	Patients showing clinical signs/symptoms	Patients showing clinical signs/symptoms	All patients	Patients showing clinical signs/symptoms
21	10925212223	Patients showing clinical signs/symptoms	Patients showing clinical signs/symptoms	Patients showing clinical signs/symptoms	Patients showing clinical signs/symptoms
22	10783643240	Patients showing clinical signs/symptoms	Whenever patient is bed ridden	All patients	Patients showing clinical signs/symptoms
23	10783242843	Patients showing clinical signs/symptoms	All patients	When patient or relatives request	Patients showing clinical signs/symptoms

PID	Respondent ID	What challenges do you face with people suffering from non-communicable diseases?					
		Challenge 1	Challenge 2	Challenge 3	Challenge 4	Challenge 5	Challenge 6
1	12544143775	No special testing at laboratories	Lack of expertise to support the patients	Shortage of advanced equipment	Poor information dissemination about NCDs	Shortage of NCDs screening tools and equipment for tests	Unavailability of affordable screening services
2	12544113622	late presentation of patients	cash crisis				
3	12544029019	money issues	patients in denial phase				
4	12544010117						
5	12543877277	Shortage of screening tools and equipment for testing	Unavailability of advanced equipment at the hospital	Lack of knowledge on NCDs by the general population	No clear guidelines for NCD patients	Poor funding from government and development partners	
6	12543851712	Unavailability of affordable screening services	Religious and traditional beliefs which cause bad health seeking behaviour	Poor funding from the government	Drugs are unaffordable for some patients		
7	12543753377	Lack of medicines	Unavailable complex lab equipment for diagnosis				
8	12543682104	Financial constraints on patients					
9	12542391758						
10	12542166298	Lack of cooperation from patients	Expensive lab tests and treatment procedures				
11	12542152626	massive knowledge gaps	not able to pay their medical bills				
12	11045742735	No clear guidelines for Zimbabwe patients	Lack of availability of newer better drugs	No dietary guidelines for our local context			
13	10946759234	Noncompliance with lifestyle changes					
14	10945142064	Financial constraints	Unavailability of drugs	Unavailability of equipment for tests	Shortage of specialists	Poor information dissemination	A lack of basic knowledge by the populace
15	10942266125	Defaulting medications	Poor monitoring				
16	10941662096	Shortage of screening tools	Traditional belief which cause bad health seeking behaviour	Lack of medications	Late presentations	Lack of investigations for follow ups	
17	10940526824						
18	10940266038	Less screening	Unavailable medication	Denial	Lack of awareness		
19	10939898917	Expensive medications	Unavailability of affordable screening services				
20	10925246304	Non compliance to treatment.	Drugs are expensive so default treatment.	No money for laboratory tests	Religion	Poor knowledge on condition.	
21	10925212223	Non availability of diagnostic	Lack of resources	No medications	No adequate manpower	No food for them	Lack of communication
22	10783643240	lack of cooperation with patients	No drugs	Lack of government support	Lack of expertise to support	No special testing at Laboratories	Lack of funding from Govt
23	10783242843						

PID	Respondent ID	In your opinion, what are the gaps hindering maximum care delivery for NCDs?				
		(i)	(ii)	(iii)	(iv)	v
1	12544143775	poverty and economic challenges	varied ways doctors used to manage different NCD patients	patients ignorance about NCDs fatality	Poor clinical care seeking behaviour by patients	Religious beliefs hindering seeking of medical care
2	12544113622	poverty	lack of government intervention			
3	12544029019	knowledge gaps on the part of the care recipients	late presentations			
4	12544010117					
5	12543877277	Economic challenges and poverty	Lack of a standard screening and treatment protocol for clinical care givers	Different approaches to NCD management at different clinics and hospitals		
6	12543851712	Poverty and economic challenges	Knowledge on NCDs management	Lack of a standard treatment framework for NCD management.		
7	12543753377	Poor financial support	Unavailable tests			
8	12543682104	Not enough resources				
9	12542391758					
10	12542166298	No clear guidelines for patient care	Expensive procedures			
11	12542152626	medicines supply chain inadequacy	government policies			
12	11045742735	Poverty and economic instabilty	Different why of managing ncds by different doctors			
13	10946759234	Lack of commitment by patients				
14	10945142064	Lack of advocacy by stakeholders	Economic conditions	Religious beliefs	Competing health providers	Poor health policies with no clear and unmeasurable outcomes or expectations
15	10942266125	Lack of public awareness	Limited resources			
16	10941662096	Few of awaress programs on preventive strategies	Lack of medicines	Lack of laboratory support in monitoring	High doctor to patient ratio	
17	10940526824	they are neglected				
18	10940266038	Lack of awareness	Lack of resources			
19	10939898917	Lack of adequate screening services	Expensive treatment options	Lack of awareness on NCDs in the community		
20	10925246304	Non availability of follow up tests	Expensive cancer drugs	Expensive laboratory tests.		
21	10925212223	Lack of medication	Lack of specialization	Lack of knowledge	Lack of medicine	Non availability of support staff
22	10783643240	Knowledge deficit in patients	Non compliance to medication	Lack of donor funding	Crowded outpatients	Lack of medicines
23	10783242843					

PID	Respondent ID	From the gaps identified above in (5), briefly state possible mitigatory measures that can be implemented.				
		(i)	(ii)	(iii)	(iv)	v
1	12544143775	Mandatory hospital-based tutoring for the public about NCDs	NCDs Educational awareness campaigns targeting high risk social classes	Free NCD treatment at designated hospitals	Availing of funding from central government and development partners	Integration of NCDs health provision with other clinical cares
2	12544113622	free health care	government bailouts for struggling patients			
3	12544029019	availing alternative funding sources	educating communities on NCDs			
4	12544010117					
5	12543877277	Integrative treatment of NCDs with other conditions	Encouraging regular NCD screening for all patients	Provision of free medication for NCDs.	Educational campaigns	
6	12543851712	Educational awareness campaigns	Free treatment at designated hospitals and clinics	Institutional policy on NCD management	Improved funding of intervention measures for NCD management.	
7	12543753377	Donor support				
8	12543682104	Government must intervene with budgeting towards NCD conditions				
9	12542391758					
10	12542166298	national medical aid scheme to assist the poor	A clear treatment guideline			
11	12542152626	Educate the communities on NCDs	Source more donor funding to cover financial gaps			
12	11045742735	Mandatory Hospital based tutoring	Availability of drugs at health institutions			
13	10946759234	Educational awareness campaigns				
14	10945142064	Better information dissemination through media	Better policy formulation	Integration of health provision	Training specialist	Decentralized care to all corners of the country
15	10942266125	Health awareness campaigns				
16	10941662096	Increase awareness programs in the community	Set up specific ncd clinics and help support them by stocking adequently and provide lab support	Decentralization of NCD clinics to district hospitals		
17	10940526824					
18	10940266038	Teach the population	Source funds for treatment	Screening tests on each clinic visit		
19	10939898917	Policy that speaks to NCDs	Affordable Health Insurance Scheme	Workplace and Community Screening initiatives		
20	10925246304	Free drugs	Free tests for Ncds	Free treatment at designated hospitals.	Supply of glucotests and insulin for free.	
21	10925212223	Training	Availing resources			
22	10783643240	teach communities	give flexible timetables	apply for funding	create a smooth flow of care	procure medicines in time
23	10783242843					
